# Repurposing of aging‐preventive pharmaceuticals to delay Alzheimer's disease progression.

**DOI:** 10.1002/alz70859_102599

**Published:** 2025-12-25

**Authors:** Andrea Mosqueda Solis, Simone Tambaro, Bowen Tang, Thais Lopes De Oliveira, Chenxi Qin, Alan Kavsek, Arvid Sjölander, Kristina Johnell, Dorota Religa, Sara Hägg, Christian Riedel, Per Nilsson

**Affiliations:** ^1^ Karolinska Institutet, Solna Sweden; ^2^ Division of Neurogeriatrics, Dept of NVS, Karolinska Institutet, Solna, Stockholm Sweden; ^3^ Karolinska Institutet, Huddinge Sweden; ^4^ Division of Clinical Geriatrics, Center for Alzheimer Research, Department of Neurobiology, Care Sciences and Society, Karolinska Institutet, Stockholm Sweden; ^5^ Theme Inflammation and Aging, Karolinska University Hospital, Stockholm Sweden; ^6^ Department of Medical Epidemiology and Biostatistics, Karolinska Institutet, Solna Sweden

## Abstract

**Background:**

Aging is the main risk factor for Alzheimer’s disease (AD). Several approved drugs currently used to treat conditions such as high blood pressure and diabetes may also influence the aging process, holding significant potential to delay AD onset or its progression.

**Method:**

Longitudinal samples from a Swedish twin cohort (N=672) were used to assess the effects of commonly prescribed medications on biological aging. Causal inference methods were further applied on genotype and National Register data to search for drug candidates with preventive effects on AD and dementia. The identified classes of compounds were evaluated for their ability to prevent aging and thereby extend lifespan in *C. elegans*, for their ability to defer paralysis and uncoordinated movement in *C. elegans* models expressing amyloid beta (Aβ) and tau, for their ability to lower Aβ levels in neuroblastoma SH‐SY5Y cells, and for their ability to ameliorate AD‐associated pathologies in *App^NL‐F^* knock‐in mice and human tau knock‐in mice.

**Results:**

We found that use of calcium channel blockers (CCBs) was associated with about a two‐year decrease in multiple epigenetic clocks (DNA methylation ages) and functional aging. Moreover, genetic variants in the glucagon‐like peptide 1 (GLP‐1) gene were associated with lower risk of developing AD, and patients on GLP‐1 analogue treatment had a lower risk of dementia. Several of the clinically used CCBs and GLP‐1 analogues significantly increased the life span in *C. elegans* and decreased levels of secreted Aβ from SH‐SY5Y cells. Exposing *C. elegans* to these compounds throughout adulthood didn’t suppress the Aβ‐induced paralysis. However, several CCBs and GLP‐1 analogues had the ability to delay uncoordinated movements in the *C. elegans* tau model.

**Conclusion:**

*Further perspectives*: Next steps include treating *App^NL‐F^* knock‐in mice and human tau mice with the most promising drug candidates. Biochemical and histological analyses, including immunohistochemistry, will be employed to assess reductions in Aβ and tau pathologies, neuroinflammation, and synaptic alterations. This work explores the potential of repurposing aging‐preventive drugs for the prevention or delay of Alzheimer’s disease, potentially creating a new strategy to target AD.

